# Systemic Mastocytosis and Essential Thrombocythemia: Case Report and Literature Overview

**DOI:** 10.3390/medicina55090528

**Published:** 2019-08-25

**Authors:** Mauro Cancian, Elisabetta Cosi, Marco Pizzi, Sandro Giannini, Irene Bertozzi, Fabrizio Fabris, Maria Luigia Randi

**Affiliations:** 1 First Medical Clinic, Department of Medicine, University of Padova, I-35128 Padova, Italy; 2 Surgical Pathology and Cytopathology Unit, Department of Medicine, University of Padova, I-35128 Padova, Italy

**Keywords:** mastocytosis, thrombocythemia, osteoporosis, hydroxyurea, zolendronic acid

## Abstract

Mastocytosis is a rare disease in which heightened amounts of mast cells accumulate in the skin, bone marrow, and other visceral organs. Upon activation, mast cells release a wide variety of preformed or newly synthesized mediators which can induce allergic symptoms and inflammatory reactions. Mastocytosis is diagnosed by biopsy and can be divided into cutaneous and systemic mastocytosis (SM). The first one affects the skin and is relatively benign, whilst SM, which involves bone marrow and other organs, may be aggressive and associate with both myelodisplastic and myeloproliferative diseases. Here we present a case of SM associated with essential thrombocythemia and complicated by severe osteoporosis, successfully treated with hydroxyurea, low-dose aspirin and zolendronic acid.

## 1. Introduction

Systemic mastocytosis (SM) is a rare hematopoietic disorder, which has been included by the World Health Organization (WHO) within the category of myeloproliferative neoplasms (MPN) [1]. The abnormal expansion of clonal mast cells (MC) observed in this disease results in a heterogeneous clinical picture, ranging from indolent SM, where patients have a normal or almost normal life-expectancy, to aggressive MC neoplasms with poor prognosis and short survival time due to multi-organ involvement [2,3].

Moreover, it is remarkable that in about 30% of patients systemic mastocytosis is associated with a further clonal, non-MC lineage disease (SM-AHNMD) [4]. Among these forms, only 15 cases of SM associated with essential thrombocythemia (SM-ET) have been published so far, many of which have incomplete information about the histologic picture and epigenetic or MPN-related mutations. Furthermore, none of these cases had been diagnosed according to the recently updated WHO criteria [5].

We present a case of SM-ET, associated with skin lesions and fulfilling WHO diagnostic criteria, that also showed evidence of severe osteoporosis. The coexistence of different conditions strengthens the relevance of a multidisciplinary approach in patients with SM, especially in those with multi-organ involvement and co-morbidities [6].

## 2. Case Report

**Figure 1:**
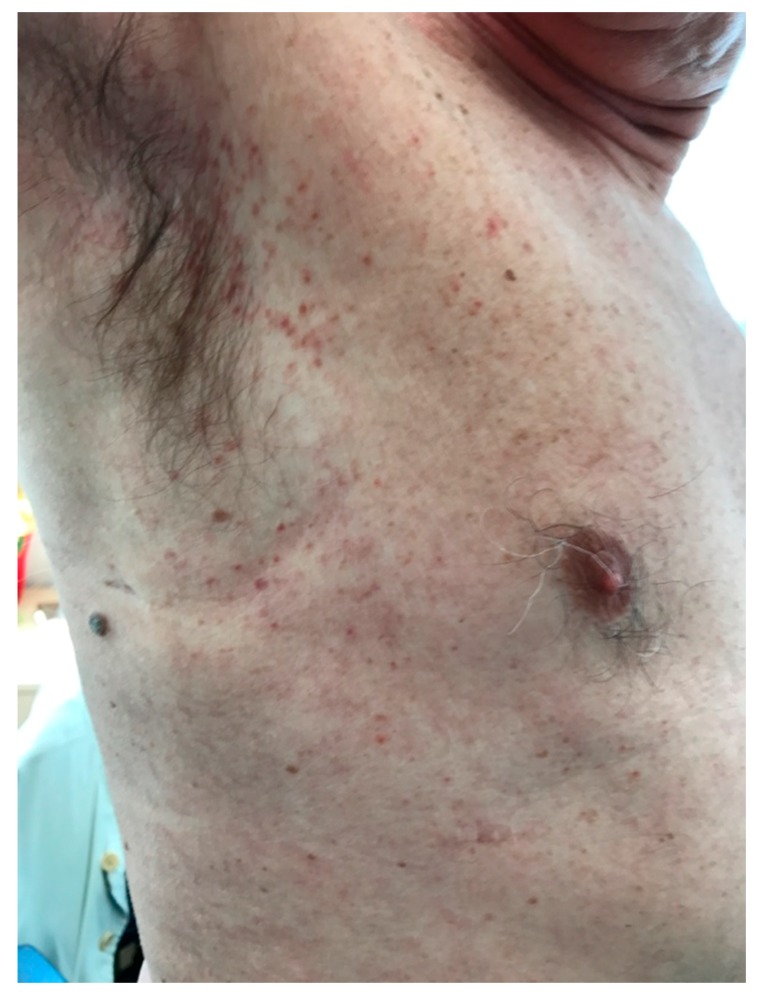
Maculopapular skin lesion, affecting mainly arms and trunk.

A 62-year-old male patient presented with longstanding thrombocytosis (600–750 × 10^9^ Plts/L) and hepato-splenomegaly associated with cutaneous papules on the trunk and arms in the last two years. Skin lesions (Figure 1), which were partially responsive to sun exposure or phototherapy, were similar to those usually observed in urticaria pigmentosa (although without the itching). On physical examination there was no evidence of lymphadenopathy, nor were any other clinically relevant findings appreciable.

The subject gave his informed consent for inclusion before he participated in the study. The study was conducted in accordance with the Declaration of Helsinki, and the manuscript submitted to Medicina was notified to the Ethics Committee (EC) of the General Hospital-University of Padova, Italy (EC protocol number 49809-25/07/2019) The patient gave consent to data collection for scientific purposes and publication.

Blood analysis confirmed a high platelet count (703 × 10^9^ Plts/L), with normal hemoglobin levels (162 g/L), white blood cell (WBC) number (8.06 × 10^9^ WBC/L) and differential count. Thereafter, the patient underwent diagnostic screening for MPN. No potential cause of reactive thrombocytosis was found, whilst abdominal ultrasound revealed an enlarged spleen (length: 18.7 cm) and *JAK2*V617F mutation (allele burden: 9.3%) was detected.

**Figure 2:**
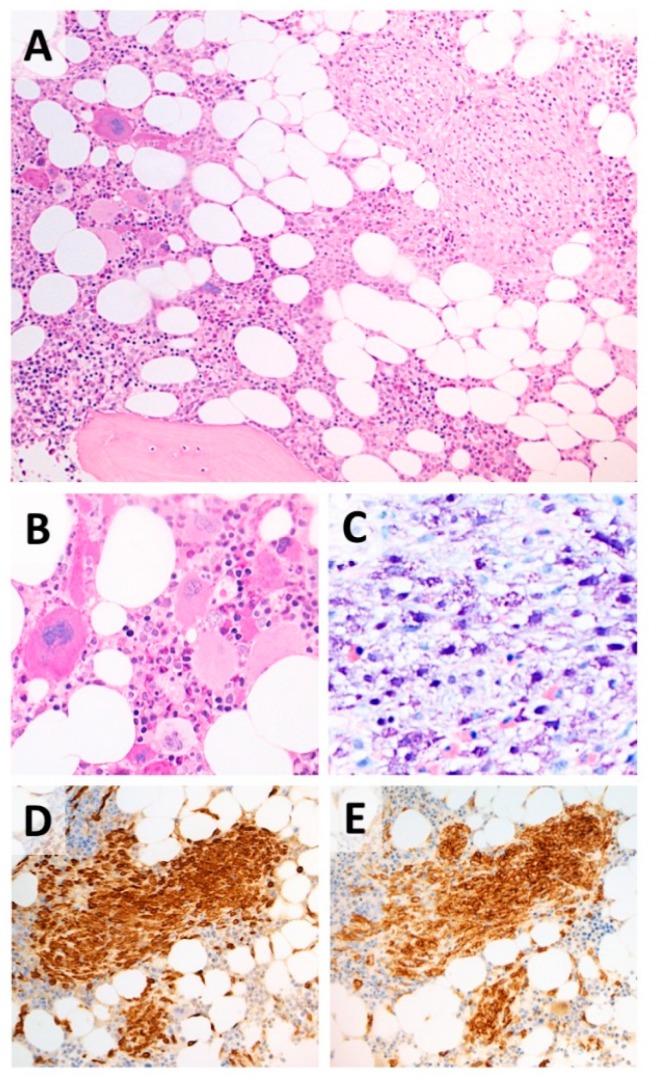
Bone marrow biopsy with histological features of SM-AHNMD. (**A**) Co-existence of non-paratrabecular nodules of atypical, spindle-shaped mast cells (right half) and clusters of large megakaryocytes (left half). (**B**) High power view of the MPN-like areas, with loose clusters of large megakaryocytes suggestive of essential thrombocythemia. (**C–E**) Spindle-shaped mast cells showing metachromatic granules at Geimsa stain (**C**), immunohistochemical positivity for triptase (**E**) and aberrant expression of CD25 (**E**). Hematoxylin & Eosin, Giemsa and Immunoperoxidase stain; original magnification ×10, ×20 and ×40). SM-AHNMD = systemic mastocytosis associated with clonal non-mast cell lineage disease; MPN = myeloproliferative neoplasm.

Bone-marrow biopsy was then performed, showing evidence of normocellular marrow, as well as of normal maturation and myeloid/erythroid ratio (3/1). However, numerous loose clusters of large/giant megakaryocytes (6–7/high resolution field) with hyper-segmented nuclei were present, together with small perivascular foci of spindle-shaped, tryptase-positive mast cells with uniformly scattered, fine metachromatic granules. Intermingled eosinophils were also found close to the MPN-like areas, where only a mild increase in reticulin fibers was detectable (Figure 2). On the contrary, interstitial fibrosis was present in MC zones (myelofibrosis grade II). Mast cells and 3% of myeloid precursors expressed CD117, whereas aberrant expression of CD25 was observed in malignant MC. The search for karyotype alteration and TET2, ASXL1, SRSF2, RUNX1 and CBL by next generation sequencing were negative.

Based on these findings, we prescribed low-dose aspirin and hydroxyurea, reassessed medical history and planned further biochemical analyses.

The patient confirmed that he had never suffered from any kind of allergy nor symptoms suggestive of mast cell activation such as urticaria, angioedema, asthma or anaphylaxis. As expected, IgE plasma levels were within the normal range (86 kU/L) and a broad screening for specific IgE (Immuno Cap ISAC^®^, Phadia, Uppsala, Sweden) was negative.

On the contrary, elevated serum alpha-tryptase concentration (123 ng/mL) and the c-KIT D816V somatic mutation were detected.

Moreover, the patient reported a previous episode of intense pain, worsened by movements and only partially relieved by rest, after very light back trauma without symptoms and signs of radicular involvement. Bone metabolism markers were assessed, and we found borderline parathyroid hormone level (62.5 pg/mL) and a slight vitamin D deficiency (25-OH-vitamin D = 50.1 nmol/L) with normal serum calcium and phosphate, bone-specific alkaline phosphatase, X-Cross-laps, 24 h urine calcium and phosphate concentrations. Multiple vertebral fractures (T7, T8, L1, L3, L5), of moderate degree and with prevalence of biconcave shape, were detected by spinal radiographs. Bone mineral density (BMD) of the lumbar spine (T-score: −3.8 SD, BMD: 0.678 g/cm^2^), as well as total femur and femoral neck measurements, were also suggestive of osteoporosis. To prevent future fragility fractures, intravenous zoledronic acid (5 mg) was therefore administered with no adverse effects.

After around three years of therapy, patient condition is good, with stable BMD, no onset of MC-related symptoms and a well-controlled platelet number (448 × 10^9^ Plts/L at last follow up).

## 3. Discussion

Myelodysplastic syndromes, acute myeloid leukemia and chronic myelomonocytic leukemia have been reported to be the hematological diseases most commonly associated with SM [7], whilst classical or unclassifiable myeloproliferative neoplasms have been seldom described as co-morbidities. At our knowledge, only 15 cases of SM associated with essential thrombocythemia have been published so far, seven of which are described in detail in Table 1 [8,9,10] (lesions of the osseous spine were found in four patients, urticaria pigmentosa in two and splenomegaly only in one). As in our case report, SM was diagnosed in one patient during screening for thrombocytosis [8]. All these patients showed a good outcome.

The present case report of SM-ET is the first one diagnosed according to the recent revision of WHO diagnostic criteria for both ET and SM [5], as the four major criteria for ET (platelet count ≥ 450 × 10^9^/L, typical bone marrow histology, presence of *JAK2* V617F mutation and absence of criteria for other myeloproliferative neoplasms) were all present together with one major criterion (compact-multifocal MC infiltrates in the bone marrow) and three minor criteria (aberrant expression of CD25 on MC, abnormal spindle-shaped morphology and *c-KIT* D816V somatic mutation) for SM.

Although SM-AHNMD has been suggested to reduce life expectancy, mainly in patients over 60 years of age [11], it should be considered that ET—the less aggressive form of MPN—does not shorten survival [12].

Therapy for SM-AHNMD is focused on treating the associated hematologic disorder, aiming simultaneously to control the symptoms of mastocytosis [3,13]. Hydroxyurea is the first-choice drug for ET patients at high risk of thrombosis [14], i.e., those with a previous thrombotic event and/or over 60 years of age, and it may also be used in patients with SM-AHNMD. This drug, indeed, was shown to be effective in five out of the seven subjects with SM-ET described in Table 1 [8,9], including our patient.

Finally, our case report confirms that annual infusion of zolendronic acid may help to reduce bone turnover and prevent vertebral fractures, which occur in 20–40% of patients with systemic mastocytosis [15,16].

## 4. Conclusions

Although the association of systemic mastocytosis with clonal, non-MC lineage disease might suggest a worse prognosis for SM, this does not seem to be the case with SM-ET. However, the ET-related risk of thromboembolism, the increased incidence of bone fractures due to concomitant osteoporosis, and the potential occurrence of severe allergic symptoms elicited by MC degranulation should be always taken into account and addressed by personalized therapy.

## Figures and Tables

**Table 1:** Clinical and molecular data in our case report of SM-AHNMD and in the seven previously published cases.

Sex/Age	Platelets (× 10^9^/L)	Clinical Findings at Presentation	Molecular Biology	Treatment	Outcome	Ref.
F/55	900	Bone lesions	-	None	Stable	[8]
M/64	900	Superficial adenopathy	-	HU	No SM (3 years)	
F/22	1000	Maculo-papular skin lesions	-	HU	No SM (3 years)	
M/60	1000	Bone lesions	-	HU	Normal Plts. Stable (6 years)	
M/68	940	Mild SM	-	HU	Normal Plts. Stable (3 years)	
F/65	1373	Maculo-papular skin lesions Bone lesions	-	HU	Normal Plts.	[9]
F/31	820	Mild spleen enlargement	*JAK2* V617F *cKIT* D816V	IFN-a	Normal Plts. Stable BM (3 years)	[10]
M/62	640	Maculo-papular skin lesions Bone lesions Mild spleen enlargement	*JAK2* V617F *cKIT* D816V	HU zoledronic acid	Normal Plts. Stable (3 years)	Present case

SM = systemic mastocytosis; SM-AHNMD = SM associated with clonal non-mast cell lineage disease; HU = hydroxyurea; IFN-a = interferon-a; BM = bone marrow histology; Plts. = platelets.
